# Fatigue and vigilance in medical experts detecting breast cancer

**DOI:** 10.1073/pnas.2309576121

**Published:** 2024-03-04

**Authors:** Sian Taylor-Phillips, David Jenkinson, Chris Stinton, Melina A. Kunar, Derrick G. Watson, Karoline Freeman, Alice Mansbridge, Matthew G. Wallis, Olive Kearins, Sue Hudson, Aileen Clarke

**Affiliations:** ^a^Division of Health Sciences, Warwick Medical School, University of Warwick, Coventry CV4 7AL, United Kingdom; ^b^Department of Psychology, University of Warwick, Coventry CV4 7AL, United Kingdom; ^c^Cambridge Breast Unit and National Institute for Health and Care Research (NIHR) Cambridge Biomedical Research Centre, Cambridge University Hospitals NHS Trust, Cambridge CB2 0QQ, United Kingdom; ^d^Screening Quality Assurance Service, National Health Service (NHS) England, Birmingham B2 4HQ, United Kingdom; ^e^Peel and Schriek Consulting Limited, London NW3 4QG, United Kingdom

**Keywords:** breast cancer, cancer screening, human behavior, vigilance decrement

## Abstract

An abundance of laboratory-based experiments has described a vigilance decrement of reducing accuracy to detect targets with time on task, but there are few real-world studies, none of which have previously controlled the environment to control for bias. We describe accuracy in clinical practice for 360 experts who examined >1 million women’s mammograms for signs of cancer, whilst controlling for potential biases. The vigilance decrement pattern was not observed. Instead, test accuracy improved over time, through a reduction in false alarms and an increase in speed, with no significant change in sensitivity. The multiple-decision model explains why experts miss targets in low prevalence settings through a change in decision threshold and search quit threshold and propose it should be adapted to explain these observed patterns of accuracy with time on task. What is typically thought of as standard and robust research findings in controlled laboratory settings may not directly apply to real-world environments and instead large, controlled studies in relevant environments are needed.

## Significance

For over 70 y, researchers have believed that as time on search tasks increases, humans make more errors detecting target “events” (and take longer): a “vigilance decrement.” Previous research has been undertaken in laboratory settings, on tasks with little control over presentation rate, but generalized to real-world scenarios, leading to regulations limiting continuous viewing time in cancer screening. We demonstrate that in a large, controlled study in clinical practice, where readers self-pace reading and rest breaks, reduced accuracy is not observed. Overall accuracy increases with time on task with fewer false alarms. Instead of limiting continuous viewing time, work environments for breast screening should allow experts uninterrupted sessions of self-chosen length, thus improving accuracy and reducing unnecessary further tests.

Errors in search and monitoring tasks have devastating consequences. In an undercover operation in airport baggage screening, operators failed to detect over 70% of mock knives, guns, and explosives (1). Expert radiologists make an average of forty million errors interpreting medical images worldwide annually (2, 3). Errors by expert radiologists substantially contribute to diagnostic error (2), which causes 80,000 deaths per year in the US (4). Across Europe, Australasia, and North America, 90,000 women each year have breast cancer missed by expert radiologists during mammography examination [0.07 to 0.15% (5–7) of >60 million women screened (8–12)] and up to 7 million women have unnecessary further tests after false-positive mammography decisions (6.5 to 12.1%) (7, 13, 14). The mechanisms of these miss errors are multiple and complex. One of the earliest and most studied proposals was an increase in miss errors over time on task, called the “vigilance decrement” (15).

The first evidence of a vigilance decrement was in radar operators in World War II, where detection of aircraft and submarines dropped after 30 to 45 min of their shift (16). This led to the seminal studies by Mackworth where Royal Air Force (RAF) observers monitored a specialized clock hand for a “signal” (the hand jumping forward two positions, rather than one) over a period of 2 h (15, 17, 18). Observers again showed a drop in performance after 30 min, prompting the authors to recommend that shifts should be limited to 30 min for tasks that require constant vigilance. A large body of literature from the field of psychology has used similar abstract tasks to determine the circumstances under which vigilance decrements are of the greatest magnitude [i.e., when event rates are high and/or successive discriminations are required (19)]. Explanations are broadly based on cognitive overload and underload (19, 20). The vigilance decrement has been observed in a range of experiments that approximate real-world activities, e.g., airport baggage screening (21), assembly line inspection (22), driving (23), radar operation (24), and interpretation of medical images (25). However, these studies lack ecological validity because participants know that they are taking part in research so the jeopardy of missing a cancer or allowing dangerous items onto an aeroplane is absent. Indeed, there is evidence from medical imaging that performance during experimental studies is not reflective of performance in clinical practice (the laboratory effect) (26–28). Despite the limitations of current research, procedures aimed at reducing vigilance decrements have been developed and implemented (29–31). These include regulations for regular breaks in cervical screening (breaks every 10 to 15 min) (30) and limiting continuous viewing in airport baggage security (breaks every 20 to 30 min) (31).

Appropriate health and safety guidance for safety-critical monitoring tasks relies on knowing whether the vigilance decrement contributes to miss errors or is simply a product of the research methods used in previous studies (32). This requires evidence from real-world studies where laboratory-effect biases are not present. Such research is challenging, because these tasks often involve searching for rare targets, which require very large studies with designs that do not interfere with safety-critical tasks. Despite a wealth of research on vigilance decrement (608 studies indexed in Medline), only four real-world studies have investigated this, two in radar surveillance (16, 33), one in baggage security (34), and one in breast cancer screening (35). In the first radar surveillance study, a brief report with little details of the methods, and the number of participants not reported, vigilance decrement was observed at 30 min (for radar operators searching for submarines) and 45 min (for radar operators searching for aircraft) (16). In the second radar surveillance study, no vigilance decrement was observed amongst 16 radar operators, in which simulated data were mixed into live air traffic data (33). In the baggage security study, x-ray screeners were divided into two groups: one who screened for 20 min (i.e., their usual working conditions) and one who screened for up to 60 min but could decide to take a break (34). Simulated threats were added to the images., which is standard practice in airport security. No difference was observed in the percentage of correctly detected images of simulated threat items between the two groups. In the breast cancer screening study, observational data on the interpretation of mammograms from 610,104 women read by 148 radiologists were extracted from the Norwegian Breast Cancer Screening programme (35). The effect on reader performance of the position of an image (ranging from 10th image to 300th image) within a batch was assessed, where a batch was defined as a reading sequence that lasted until a break of 15 min or more occurred between two interpretation decisions. There was some evidence for a vigilance decrement shown as a small but statistically significant reduction in true positive (cancer detected) interpretations with time on task (0.2/1,000 decrease over first 100 cases, 5% relative to first 10 cases), but this study excluded missed (interval) cancer so could not assess test sensitivity. There was also a concurrent larger decrease in false positive recalls (10/1,000 decrease over first 100 cases, 19% relative to first 10 cases). However, these are purely observational data with no analysis of whether this is driven by a radiologist’s vigilance decrement or confounding factors such as radiologists moving more difficult cases for later consideration or women who are more likely to have cancer being allocated to the first half of batches.

A large randomized controlled trial of >1 million women attending the English mammography screening programme investigated an intervention which changed the order in which cases were examined to reduce the impact of the vigilance decrement (36). The intervention had no effect in short work sessions (37). These data have the advantage of bespoke trial software to detect and correct for potential confounding such as radiologists moving cases, and the intervention reversed case order enabling analysis of potential systematic biases in risk of cancer with batch position. In the current work we use data from that study to determine whether experts examining breast screening mammograms for signs of cancer experience a vigilance decrement, in both short and long work sessions.

## Results and Discussion

We studied breast screening mammograms from 1,069,566 women (mean age 59 y), of whom 226,506 (21%) were attending their first-ever screening appointment. Each woman’s mammograms were independently examined for signs of cancer by two qualified specialist experts (henceforth referred to as “experts”). In all, 360 experts are included in this study. Then, 8,761 (0.82%) of the women had cancer detected at screening and a further 2,046 (0.21%) had cancer detected symptomatically within 3 y of screening. Further descriptive statistics appear in *SI Appendix*, Fig. S1 and Table S1.

### Mammography Speed and Accuracy Improve with Time on Task.

**Fig. 1.**
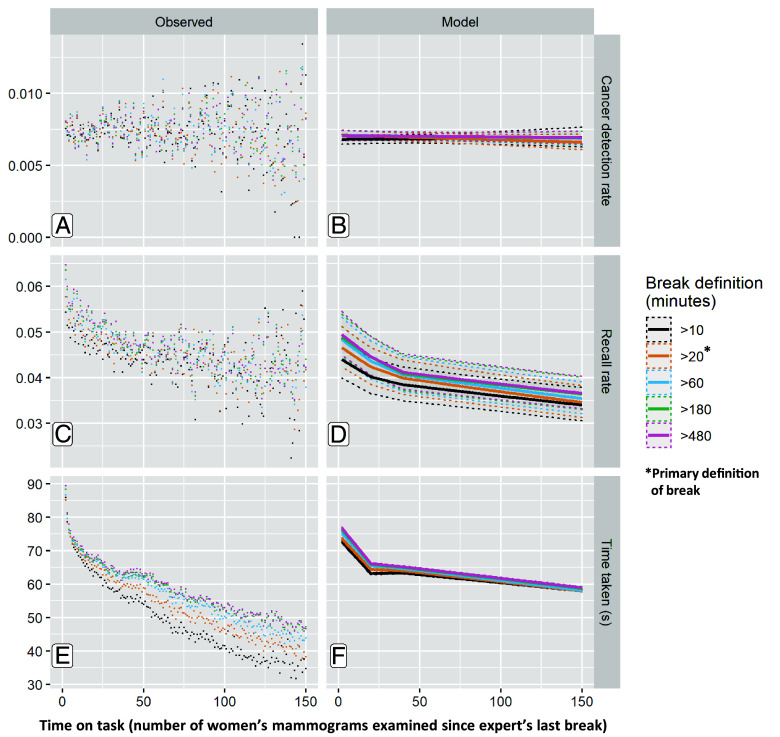
Performance metrics with time on task, represented by the number of women’s mammograms examined consecutively since the expert’s last break. Observed values are shown to the *Left* and multilevel model fitted values (shown as a solid line with 95% CIs as dotted lines) to the *Right*. Models were adjusted for women’s age and whether they have previously attended screening, with clustering for expert and screening center. The primary definition of a break was 20 min without inputting a decision on the computer. Sensitivity analyses exploring different definitions of what constituted an expert’s break: 10, 60, 180, or 480 min are shown in different colors. (*A*) Cancer detection rate data calculated as the proportion of women that were correctly identified as having cancer (*B*) modeled result; cancer detection rate; (*C*) recall rate data (calculated as the proportion of women the expert indicated required recall for further tests) (*D*) modeled recall rate result; (*E*) mean time taken to examine each woman’s mammograms data; and (*F*) modeled mean time. For example, an orange data point with x = 50 includes data from every woman who was examined at the 50th position in the session after a break of 20 min or more. If the definition of a break is changed to at least 60 min, shorter breaks are ignored, and some women are re-categorized into larger sessions for analysis (blue). Because of this re-categorization for sensitivity analyses of different break definitions, women’s mammograms can appear more than once as represented in the different colored analyses but only once in each color.

**Fig. 2.**
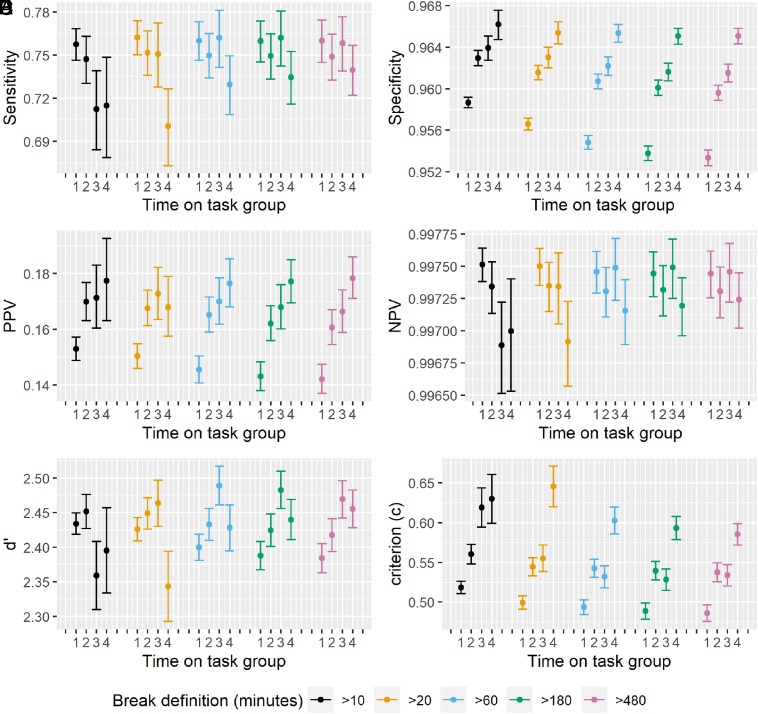
Test accuracy represented by (*A*) sensitivity, (*B*) specificity, (*C*) PPV, (*D*) NPV, (*E*) d-prime (*F*) criterion (decision threshold/willingness to recall women for further tests), with time on task. Bars represent 95% CIs. Data in orange represent the primary analysis using a break definition of 20 min. Time on task is represented by how many women’s mammograms have been examined with a 20-min break. Data were analyzed in 4 groups based on the position number of mammograms in a session: group 1—mammograms that were examined at the 2nd to 30th position in the session after a break of at least 20 min, group 2—mammograms that were examined at the 31st to 60th position in the session after a break of at least 20 min, group 3—mammograms that were examined at the 61st to 90th position in the session after a break of at least 20 min, group 4—mammograms that were examined at the 91st to 200th position in the session after a break of at least 20 min. Sensitivity analyses exploring alternative definitions of break length are shown in different colors.

The vigilance decrement predicts a reduction in cancer detection rate (number of cancers detected by the expert per thousand women screened) with time on task. We found that neither cancer detection rate (Fig. 1 *A* and *B*) nor test sensitivity (proportion of women with cancer who were detected by screening, Fig. 2*A*) changed over the course of examining 200 women’s mammograms since their last break of 20 min or more [Odds Ratio (OR) [5 extra women’s mammograms since the expert’s last break] = 0.998 (95%CI 0.994 to 1.0008)]. This pattern was observed for our main definition of a break (20 min or more without inputting a decision into the computer, colored orange in Figs. 1 and 2) and our sensitivity analyses defining a break as >10, >60, >180, or >480 min without inputting a decision (black, blue-green, and pink in Figs. 1 and 2, respectively). Examining each woman’s mammograms takes a median of 36 s (mean 69 s, distribution in *SI Appendix*, Fig. S5), so this translates to no vigilance decrement observed after more than 2 h on task for most experts and over 1 h for the faster experts.

Working for more than an hour without a break resulted in improvements in overall accuracy rather than decrements. This was due to a reduction in false positive recalls (false alarms where a woman without cancer is incorrectly recalled for further tests, which causes her anxiety and consumes significant resources). The overall recall rate (proportion of women recalled for further tests, using the 20-min break definition in Fig. 1 *C* and *D*) decreased rapidly from 4.66%, (95% CI 4.23 to 5.12%) when the expert started the task, to 3.99% (3.63 to 4.39%) when examining the 40th woman’s mammograms, to 3.69% (3.35 to 4.06%) when examining the 100th woman’s mammograms, and 3.24% (2.90 to 3.62%) when examining the 200th woman’s mammograms without taking a break of 20 min or more. This is clinically and operationally significant, as in a national programme screening 2 million women/year the difference between a recall rate of 3.24% and 4.66% is an additional 28,400 (44%) unnecessary false positive recalls to assessment. This was similarly reflected in increasing test specificity (proportion of women without cancer who are correctly told they do not have cancer, Fig. 2*B*) and in increasing positive predictive value (PPV) (the proportion of women recalled for further tests who have cancer, Fig. 2*C*) with time on task.

In addition to becoming more accurate, experts also made each decision more quickly as the number of women’s mammograms examined since their last break increased (a measure of time on task, Fig. 1 *E* and *F* and *SI Appendix*, Table S3). At the start, experts took a mean of 73.7s (95%CI 73.4 s to 73.9 s) to examine each woman’s mammograms. By the 20th woman’s mammograms examined since their last break of at least 20 min, this had reduced to 64.4 s (95%CI 64.1 s to 64.6 s), to 60.6 s (95%CI 60.4 s to 60.9 s) by the 100th woman’s mammograms and to 55.1 s (95%CI 54.9 s to 55.4 s) by the 200th woman’s mammograms. Full model results are in *SI Appendix*, Tables S2 and S5.

### Changes in Accuracy Are Dependent on Break Length.

After longer breaks, the experts start the session with a higher recall rate than after shorter breaks, and they decrease at a similar rate. When reading the first session of a working day following at least 8 h without reading activity (the >480-min-break definition, Fig. 1 *C* and *D*), the recall rate is initially high (5%), and reduces with time on task (3.5% at the 200th woman). When including short breaks of 20 min or more, the expert has partially reset their recall rate to be higher again (4.7%, Fig. 1 *C* and *D*, >20 min definition), and then it declines again with time on task (3.2% at the 200th woman). Similarly, specificity is lower at the start of a working day (95.3%, Fig. 2*B*, >480 definition, group 1: first 30 cases) and increases with time on task (96.5%, Fig. 2*B* group 4: cases 91 to 200). After a 20-min break or longer, specificity is reduced but not as much as at the beginning of the day (95.7%, Fig. 2*B*, group 1: first 30 cases). Time taken per case follows the same pattern of being highest at the beginning of a working day, decreasing with time on task, with breaks within a day not fully resetting to match time taken at the beginning of the day (Fig. 1 *E* and *F*). This is further explored in *SI Appendix*, Fig. S4, which shows the same patterns when breaks of <1 h, 1 to 3 h, 3 to 12 h, and >12 h are analyzed separately.

### Patterns of Increasing Accuracy Are Robust When Considering Bias and Statistical Power.

**Fig. 3.**
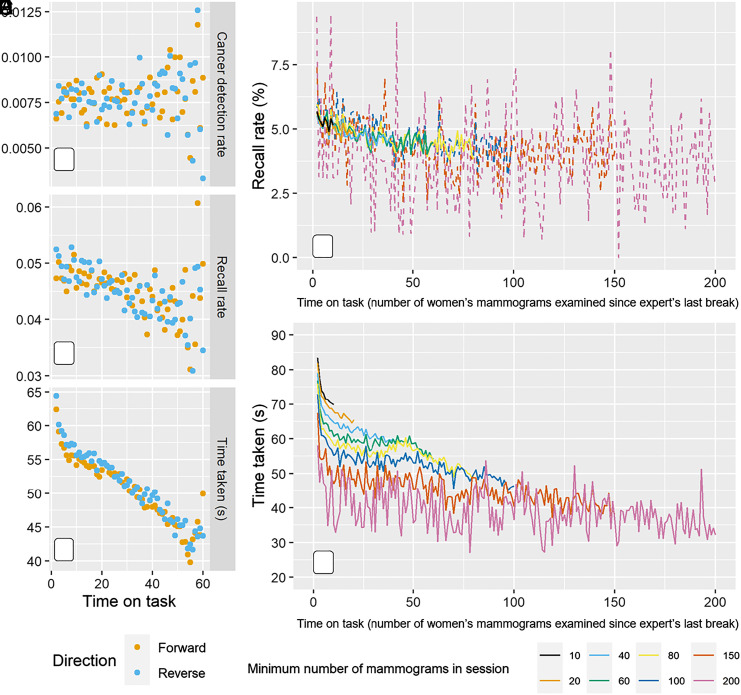
Robustness of results to individual woman level confounding (*A*–*C*) and expert level confounding (*D* and *E*). Cancer detection rate (*A*), recall rate (*B*), and speed of decision-making (mean time taken) (*C*) with time on task for the trial intervention arm, where women were organized into sessions and two experts examined them in the opposite order to one another. Data shown for 534,108 women’s mammograms in the “forward” direction and 524,908 in the “reverse” direction (session position of mammograms read in intended order, plots truncated at position 60). Recall rate (*D*) and speed of decision-making (*E*) with time on task by minimum task length. Only reading sessions longer than the minimum task length (minimum number of mammograms in session) are included to remove potential confounding by different experts spending different time on task. After 20 min without a decision input, the expert is considered to have taken a break and started a new session. Time on task represented by number of women’s mammograms examined since the expert’s last break.

These are observational data, so consideration must be given to whether these effects might be driven by measured or unmeasured confounders or biases. First, were women whose mammograms were examined first in a reading session systematically different to those examined later? Whilst the allocation system suggests no reason for this, in large well-powered datasets, it is important to examine this empirically. This was tested using data from the intervention arm of the original trial, where up to 111 women’s mammograms were grouped together in “sessions.” The first and second experts examined each session in the opposite order to one another, yet the reduction in recall rate and time taken were observed with both experts (Fig. 3 *B* and *C*). Therefore, the effects are unlikely to be due to confounding associated with the woman’s characteristics.

Confounding at the expert level was also considered, as experts themselves could choose the length of time on task since their last break. Were experts who chose to take more breaks systematically different from those who took fewer? Specifically, were experts who read long sessions quicker and have lower recall rate, and it was the amalgamation of different session lengths giving the appearance of decrease over time? Analyses were repeated for each session length, for example, only including task sessions when a given minimum number of mammograms were examined. The same effect of decreasing recall rate and decreasing time taken to examine each women’s mammograms was found for every session length (Fig. 3 *D* and *E* and *SI Appendix*, Fig. S2). This demonstrates that these effects are not caused by expert-level confounding because they were still present when fixing session length. Further, we investigated whether effects were caused by experts changing the order in which they examined the women’s mammograms, and this was also not causing confounding as shown in *SI Appendix*, Fig. S3.

### Theoretical Explanations for Changes with Time on Task.

Signal detection theory is the key framework underpinning most models for understanding performance and error in medical imaging. Within this framework, the improvement in accuracy with time on task observed here could be due to the experts’ fundamental accuracy increasing, characterized by moving to a higher receiver operating characteristic (ROC) curve (an improvement in ability to discriminate between mammograms with and without cancer), or due to a change in decision threshold (becoming more reluctant to recall cases) within the same fundamental ROC curve. Whilst overall accuracy does improve with time on task, this still may be explained by a change in decision threshold if the experts are becoming more reluctant to recall whilst operating on a low gradient part of the ROC curve (i.e., they are making very inclusive decisions to recall many women, so changing their decision threshold only excludes women with minimal signs of cancer). A previous study in the Norwegian breast screening programme did find a small reduction in true positive recalls with time on task, alongside a larger reduction in false positive recalls (35). Their programme is similar to the United Kingdom’s, but overall cancer detection rate in their study was 42 per thousand women screened, whereas in our study, it was 88 per thousand women screened. It is possible that both cohorts experienced a threshold shift with time on task, but in Norway, this affected cancer detection to a greater extent because they are operating at a different point on the ROC curve where a threshold shift will have a greater effect on cancer detection rate due to a differing slope. However, this comparison is confounded by the different population risk profiles and screening intervals.

**Fig. 4.**
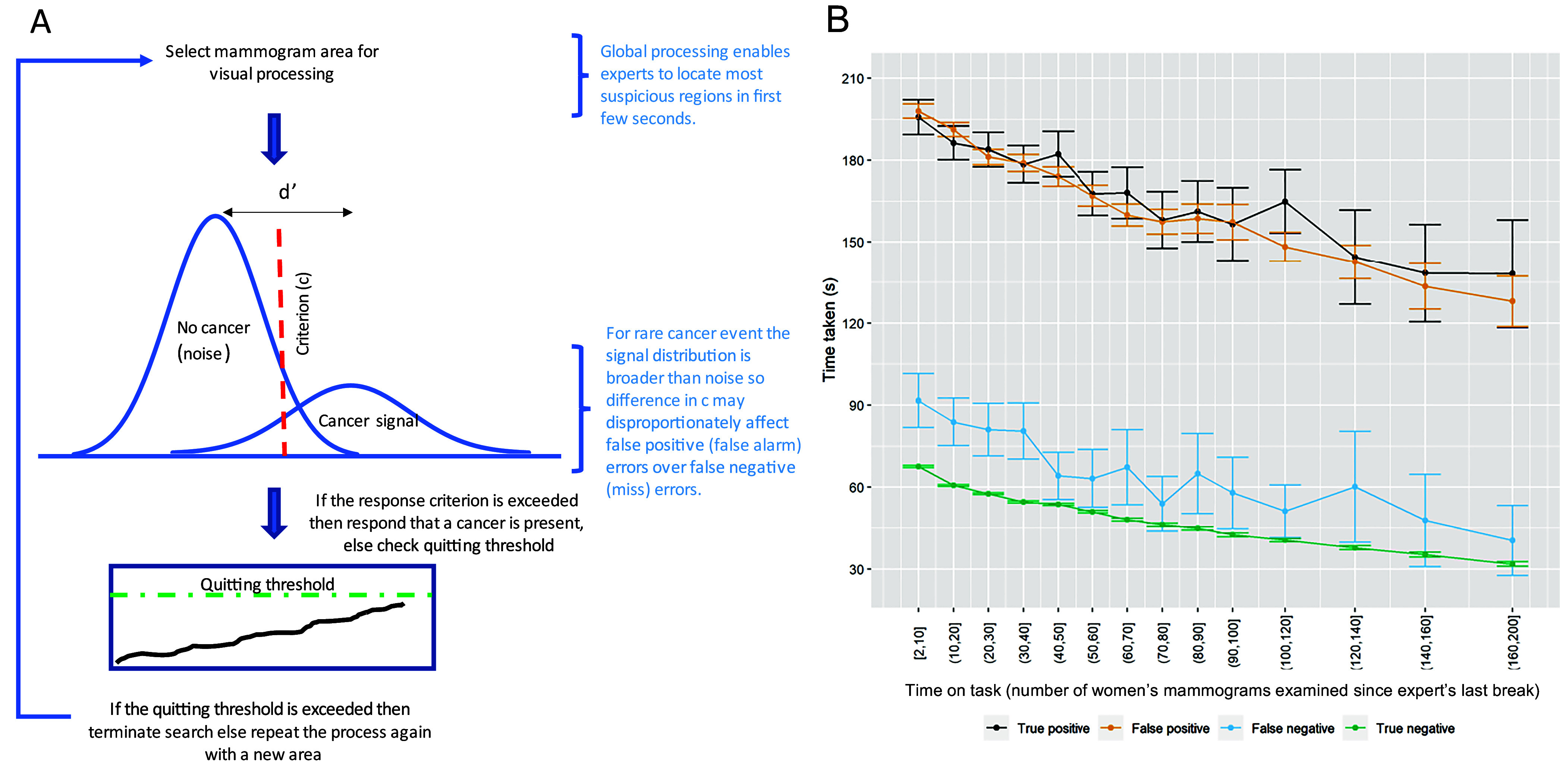
(*A*) Multiple decision model proposed by Wolfe et al. (39), with global processing (42). If the selected area contains a cancer and response falls to the right of the criterion decision line, then the cancer would be detected, else it would be missed. This process would continue until the quitting threshold is reached, at which point search would terminate. With time on task, our data suggest the Quitting threshold (denoted by the green dotted line) would be lowered, and the response threshold (denoted by the red dotted line) would move to the right (so that experts are more conservative in their response to say there is a cancer). (*B*) Our results for speed of reading (mean with 95%CI) with time on task (number of women’s mammograms examined since the expert’s last break), shown for each decision outcome (true positive, false positive, true negative or false negative outcome).

The multiple-decision model (MDM) of search (38) (Fig. 4) uses signal detection theory to explain the impact of disease prevalence on accuracy. This model proposes that at low prevalence (as found in cancer screening), experts do not change their fundamental accuracy (as measured by d-prime), but they spend less time searching the display and have increased decision thresholds (so are more willing to say that a mammogram is cancer-free) (39). We propose applying the MDM to time on task in a similar manner to low prevalence. This would predict a universal reduction in reading time due to a lowering of the “quitting threshold” for search over time (i.e., experts will spend less time searching each display before making a decision), as indicated in Fig. 1 *E* and *F*. It also predicts a change in decision threshold over time so experts would be less willing to say there is a cancer, without a corresponding change in d-prime—consistent with our signal detection data shown in Fig. 2 *E* and *F* and the increase in specificity over time as seen in Fig. 2*B*. The Criterion (decision threshold) in Fig. 2*F* may also be higher at the start of sessions preceded by a shorter break, which might indicate short breaks do not result in a full reset of decision threshold or reduction in specificity compared to long breaks. This is explored in *SI Appendix*, Fig. S4 which shows that specificity and criterion are lower after a long break of >12 h, compared to after a short break of <1 h. The mammography task is thought to consist of three stages, search (global processing then targeted search to locate regions of interest), recognition, and decision (40). There is evidence that experts can extract important global information from an image within the first glance to determine whether an image contains an anomaly or not (in some instances within the first 250 ms of an image being presented) (41, 42). This “gist” of information is likely to shape an expert’s subsequent search so that they are more effective at detecting cancer and less likely to introduce additional false positive recalls, through focusing on the most important areas of the mammogram first. In these circumstances the effect of shortening of search on the accuracy of the highly experienced experts in this study is reduced. In the Norwegian study the speed of reading increased with time on task in a similar way to our results, and their modelling indicated that 17% of the reduction in true positive results was mediated by reading speed. In the United Kingdom, time spent reading each case was longer than in Norway, which may explain why the increased reading speed did not have the same impact. Whilst this explanation is a good fit to these data, it doesn’t preclude other explanations. It does suggest that considerations of break scheduling are more complex than a simple reduction in vigilance and should also consider threshold shifts from the current threshold and related clinical outcomes.

Initial data on radar monitoring (16), and successive decades of research, show a clinically and statistically significant decrement in detection with time on task. Why do we see a different pattern in screening mammography, predominantly of improved performance? Within mammography, search for an anomaly is perceptually more varied and complex, compared to radar surveillance (and similar lab-based experiments), but perhaps most importantly, signals are not time-limited or transient in nature. Furthermore, experts may be intrinsically motivated in their goal to reduce illness (as opposed to being told by superiors/experimenters to find a target). Both of these factors are known to reduce the vigilance decrement with some suggestion that the vigilance decrement may be a byproduct of the vigilance study design (20, 32). Radiologists may also choose to look at a mammogram for as long as needed (within reason to ensure they still read their large volume of images within a batch, see *SI Appendix*, Fig. S5 for distribution of time taken per case). In contrast, tasks like radar monitoring and the original vigilance studies used stimuli where the timing of the target was controlled by the experiment rather than the reader (e.g., ref. 18). This may have an effect on the vigilance decrement. Indeed, a study involving 22 baggage screeners examining images with simulated threats at an international airport, where the timing was controlled by the expert, showed limited evidence for vigilance decrements when the task load (rate of presentation of baggage images) was low to medium but this developed as task load became high [significant interaction between task load and time on task) (34)].

Similarly, examining mammograms is time pressured due to reading volume overall but experts can determine their own break and task scheduling, and there is evidence that breaks (either enforced or self-selected) can reduce the vigilance decrement. In the laboratory, the introduction of breaks has been shown to reduce the vigilance deficit for both self-paced breaks (43) and those that are imposed [e.g., refs. 44 and 45, though this is not a universal finding (46)]. For example, Helton and Wen (47) suggest that the addition of a break can lead to a renewal of resources that would otherwise be depleted according to resource depletion theories of the vigilance decrement (see also ref. 48). As experts can regulate rest breaks more than would typically be expected in laboratory vigilance studies, this could be a factor in why we saw no vigilance decrement in our mammogram data. In fact, readers in an X-ray baggage screening task reported more engagement in the task when they could choose for themselves when to stop, suggesting that control of when to take breaks is in itself beneficial (34).

The practical purpose of understanding the underlying mechanisms is so we can generalize to other medical imaging tasks, and broader search tasks. These tasks will vary in disease prevalence, recall threshold, accuracy, expertise of experts, and reading environment. The current findings demonstrate that vigilance decrement theory should not be applied without empirical evidence in the real-world setting. Similarly, we cannot assume that the accuracy and speed improvements with time on task reported here are generalizable to other tasks requiring vigilance, though they are most likely to be generalizable to self-paced radiology and pathology tasks.

## Conclusions

We found no significant vigilance decrement experienced by qualified experts examining up to 200 women’s mammograms sequentially. Instead, performance improves with time on task through increased speed and a reduction in number of false positive recalls for assessment. Further, shorter breaks were associated with fewer false positive recalls to assessment at the beginning of the next batch compared to longer breaks. Our results suggest that population breast screening programmes should enable experts to review several sessions of up to 200 women’s mammograms consecutively if they wish, with self-selected break scheduling, minimize interruptions in the work environment. This contrasts, in part, with the results of the only other study on this topic which found a small but statistically significant decrease in sensitivity with time on task (35). Both studies found an increase in specificity and decrease in time taken per case with time on task. In combination, both of these studies support a MDM with a threshold shift to explain behavior changes with time on task, rather than a vigilance decrement.

Determining the extent to which our findings generalize to other repetitive clinical tasks such as those within other screening programmes and real-world vigilance tasks will require further applied research. However, this study demonstrates that a huge literature of laboratory-based psychological research incorrectly predicts what may happen in at least one clinical practice task—that of mammography reading. Further research is required to understand the underlying psychological mechanisms and how they impact real-world health outcomes. Laboratory and real-world research should be brought together through interdisciplinary collaborations to answer these questions.

## Materials and Methods

### Methods.

This observational study uses data from a randomized controlled trial; Changing case Order to Optimise patterns of Performance in Screening (CO-OPS, ISRCTN46603370), which is reported in detail elsewhere (37). Ethical approval was granted by the Coventry and Warwickshire National Health Service (NHS) Research Ethics Committee on June 27, 2012 (ref WM/0182). Approval to archive the data and carry out further analysis on the dataset was granted on June 7th, 2022 (ref 12/WM/018). Informed consent was at the center level by the director of breast screening, as the intervention and control group were considered both to be standard practice. Women’s mammograms were examined in sessions (median size 35 women) grouped together within the computer software, with two experts independently examining each session in either the opposite order to one another (intervention) or the same order (control). In practice, many experts examined several sessions sequentially without a break. In this study, we examined accuracy with number of women’s mammograms examined since the expert’s last break (as a proxy for time on task). We defined a break as either 10 min, 20 min, 60 min, 180 min, and 480 min without inputting a decision into the computer software. The primary definition was 20 min. The outcomes of recall rate, cancer detection rate, and time taken to read were modeled to assess the effect of number of women’s mammograms examined since a break; using screening center and expert as levels in a multilevel model.

### Study Population.

The CO-OPS trial involved 46 breast cancer screening centers in England, United Kingdom, for 1 y commencing in 2012. The practice in those centers was for two experts to independently examine women’s mammograms to decide whether to recall her for further tests. Discordant decisions were resolved by arbitration, and in some cases, decisions by both experts to recall were also arbitrated. Most centers did not blind the second expert to the decision of the first. Experts were all radiologists, breast clinicians, or radiography advanced practitioners or consultants qualified to read in the NHS breast screening programme (49), requiring a minimum of 5,000 women’s mammograms to be examined per year. Within the computer software mammograms from all women screened at a single location on the same day were presented together as a list, we refer to this as a session. The expectation was that experts would examine a whole session without a break. In practice, many experts report examining several sessions sequentially without a break. The CO-OPS trial questioned whether the second expert examining the session in the reverse order to the first expert would change the cancer detection rate, via vigilance decrements occurring for each expert at different points in the session, i.e., whilst examining different women’s mammograms. The intervention was not effective (37). The observational analysis reported here examines the patterns of performance with time on task, using the time stamp from the computer software of each expert’s decision to recall or not. Every woman screened by each center as part of the NHS Breast Screening Programme during the trial period was included in the trial and this subsequent observational analysis; with 1,194,147 women involved in this final analysis.

### Outcomes.

This study uses three main outcome variables: the proportion of women that the expert-recommended recalling for further tests (recall rate), the proportion of women in which the expert detected a cancer (cancer detection rate, requiring both the expert to suggest recall and the follow-up tests indicate biopsy-proven breast cancer), and the time taken for the expert to examine and decide whether to recall the woman. Outcome data for these three variables are complete: recall decisions and a time stamp for when they were made were automatically populated in the software and form part of workflow at the center, and cancers detected after recall from screening and proven by biopsy will be complete due to the standardized quality assurance processes. We explore the effect that time on task (characterized as number of women’s mammograms examined since a break) has on these three outcomes. We also examine test accuracy using the reference standard of biopsy-proven breast cancer either after recall from the screening appointment or symptomatically detected within 3 y after screening. Symptomatically detected cancers were communicated back to screening centers from the English cancer registry. Women who moved abroad, had cancer detected after longer than 3 y, or in whom the cancer registry failed to report back to the screening center would not have been included in this dataset. Women who did not have cancer detected at screening and did not have a record of a symptomatic cancer in the 3 y after screening were assumed to not have cancer. Screening test outcomes are defined as true positive (TP, the woman had cancer which was detected), false negative (FN, the woman had cancer which was missed), false positive (FP, the woman did not have cancer, she was worried unnecessarily after incorrect recall for further tests), and true negative (the woman did not have cancer and was correctly reassured). The standard four test accuracy metrics were calculated as follows: sensitivity = TP/(TP+FN), specificity = TN/(TN+FP), PPV = TP/(TP+FP), and negative predictive value (NPV) = TN/(TN+FN).

### Data Preparation.

The trial was implemented through adaptations to the UK National Breast Screening Service’s (NBSS) computer system, which records each expert’s identity and decision for every case alongside any subsequent arbitration decision and whether cancer was detected following recall from screening. The system also records whether cancer was detected symptomatically in the years following screening, but this requires human input through a national system based on cancer registry data, so may be incomplete. The NBSS computer system was adapted for the CO-OPS trial to record additional variables, including date and time of each decision, and to detect when cases were not read within the intended order within a session.

Experts often examine more than one session in succession and performance over time periods longer than a single session is of interest. Therefore, we used the exact time and date of each decision to combine sessions examined subsequently by the same expert into a single session, using different assumptions regarding the time period that had elapsed without a decision (and therefore assumed to represent the reader taking a break). We considered the primary definition of a break as 20 min without a decision as experts would very rarely take longer than 20 min to make and report a decision. We undertook sensitivity analyses by additional shorter or longer break time definitions (10, 60, 180, or 480 min) to check the robustness of this assumption. Unless otherwise stated, results are given for the 20-min definition.

We used cases examined as the first or the second expert to establish the case order but only used decisions made as the first expert in our analysis to preserve independence of cases in the models and to ensure decisions were made independently without consulting the other expert’s decision. We excluded cases that were not examined in the intended order. We undertook a sensitivity analysis to check whether including these moved cases changed conclusions and it did not (*SI Appendix*, Fig. S3). Data preparation is described in more detail in the methods section of *SI Appendix*.

### Statistical Analysis.

Multilevel models were used; with the individual mammogram as level one, expert as level two (to account for the effect of different individual experts), and center as level three. Generalized linear models were used; with recall and cancer detection (binary outcomes) analyzed with logistic models and time taken on the case analyzed with a gamma distribution. The main explanatory variable studied was position in session, as defined above (as a proxy for time on task), with models evaluated for each definition of session position, determined by the different break definitions. Position in session was included in the models for recall and time taken to read using a linear basis spline, with knot points at positions 20 and 40, but as a linear term for cancer detected, because the pattern for cancer detected was not curved in shape. The age of the woman at the time of the mammogram and whether it was the woman’s first mammogram (prevalent) or a subsequent one (incident) were also used as explanatory variables.

Each model was run on a subset of the dataset, removing all mammograms read either first in the session (as these may be systematically different based on our session definition) or after position 200 in the session (as these represented influential outliers). The number of mammograms removed for these reasons is different for each break time definition and is shown in *SI Appendix*, Table S1. For the models of time taken to read, mammograms for which the time taken to read was greater than 10 min (and some cases where the time was recorded as 0) were also removed from the dataset.

To examine the possibility that there was some confounding present from systematic differences in characteristics of women examined early and late in the session we undertook two extra analyses. First, we analyzed pattern of performance over the course of the session comparing the cases from the intervention trial arm, those examined in forward and those examined in reverse order by reader 1. These were sessions examined in the opposite order, so thus we separated the effect of any confounder associated with the characteristics of the women screened at different session positions with the effect of time on task. The analysis was performed using R software, with the multilevel models being fitted with the “lme4” package. Statistical significance was assessed at the 5% level.

CIs for sensitivity, specificity, PPV, and NPV were calculated using the Wilson method for binomial CIs.

The measure d′ (d prime) is calculated by d'=z(Sensitivity)d'-z(1- Specificity), and criterion by $c=-1/2\left(z(\text{Sensitivity})+z(1-Specificity)\right)$, where z is the inverse cumulative normal distribution function, as given in Macmillan and Creelman (50). The CIs were calculated using the approximation given by Gourevitch and Galanter (51).

## Supplementary Material

Appendix 01 (PDF)

## Data Availability

These data that support the findings of this study are available on reasonable request from the corresponding author [S.T.-P.], conditional upon that request meeting the requirements of the study ethical approvals. These data are not publicly available due to the inclusion of patient data. The code are available upon reasonable request from the corresponding author [S.T.-P.].
