# Supplementary material for: Fatigue and vigilance in medical experts detecting breast cancer
Source: Proc Natl Acad Sci U S A. 2024 Mar 4;121(11):e2309576121. doi: 10.1073/pnas.2309576121 (PMC10945845; doi:10.1073/pnas.2309576121)
Supplement: Supplementary file 1 — Appendix 01 (PDF) [file pnas.2309576121.sapp.pdf]

Supplementary Materials for:

Fatigue and Vigilance in Medical Experts Detecting Breast Cancer

Sian Taylor Phillips, David Jenkinson, Chris Stinton, Melina A Kunar, Derrick G Watson, Karoline Freeman, Alice Mansbridge, Matthew G Wallis, Olive Kearins, Sue Hudson, Aileen Clarke

Correspondence to: s.taylor-phillips@warwick.ac.uk

This PDF file includes:

Supplementary Text
Figs. S1 to S5
Tables S1 to S5

This supplement contains additional details for the study methods (section 1 figure S1), descriptive statistics (section 2 table S1), full model outputs for adjusted and unadjusted models (Section 3 tables S2-S5). Results for speed of reading are presented for median rather than mean, demonstrating no impact of excluding outliers on results (section 4 figure S2). Results for cancer detection rate, recall rate and speed of reading are presented both with and without cases which were examined out of the intended order, demonstrating this exclusion did not impact results (section 5, figure S3).

1. Supplementary Methods

Defining how many cases had been examined in each reading session

Experts examine mammograms in screening practice in predefined sessions. Each session consists of a full or half day for a screening mammography machine at one location. Sessions are created alphabetically by surname from lists of eligible women from general practitioners' databases. On the radiology software each session is opened separately and is displayed as a list of women for whom the expert sequentially decides whether to recall each for further tests. The computer software records any cases which were not examined in the sequential order assigned. After completing a session, experts often immediately start another session, which can be achieved simply and quickly with the click of a mouse. The primary analysis in this paper considers an expert to have kept working constantly even if switching session as long as they did not spend longer than the predefined times between making decisions for subsequent women.

To create the new sessions we put the mammograms examined by each expert into chronological order (using the date/time stamp). Individual experts read some mammograms as first expert and others as second expert. Thus, we separated the data from each mammogram into two records, one for the data from each expert. The dataset was sorted by centre, expert and date/time to give a list of cases read by each expert in chronological order

(regardless of whether it was as first expert or second expert). The difference between the time stamps of consecutive cases was calculated to give the time taken for that case by that expert.

The new sessions were then created (with new session numbers) by assigning each case to either the same session as the previous case if the time taken was less than the break definition, or to a new session if the time taken was greater than the break definition. This was done for each of the different values of the break definition (10,20, 60, 180 and 480 minutes).

It was possible that where mammograms from different original sessions have been combined into the same new session that the new session will contain some mammograms read as first expert and others as second expert. The dataset used for modelling contains all of the mammograms once, with their session position and outcomes taken from the first expert only. This also ensures that the mammograms were examined independently without consulting the other expert's decision.

The trial data included a field indicating whether the actual order that the mammogram was read in was the intended order. Mammograms that were not read in the intended order were excluded from the models, as they may be systematically different, for example occasionally difficult cases which were put aside for later review. Mammograms that were read in the first position of a new session (as defined by the different break definitions) were also excluded, as it is not possible to determine the time taken to read them.

It was also necessary to exclude data from centres where it was not possible to distinguish between different experts using the expert ID code in the dataset.

The distributions of the session position numbers used in the models are shown in Figure S1.

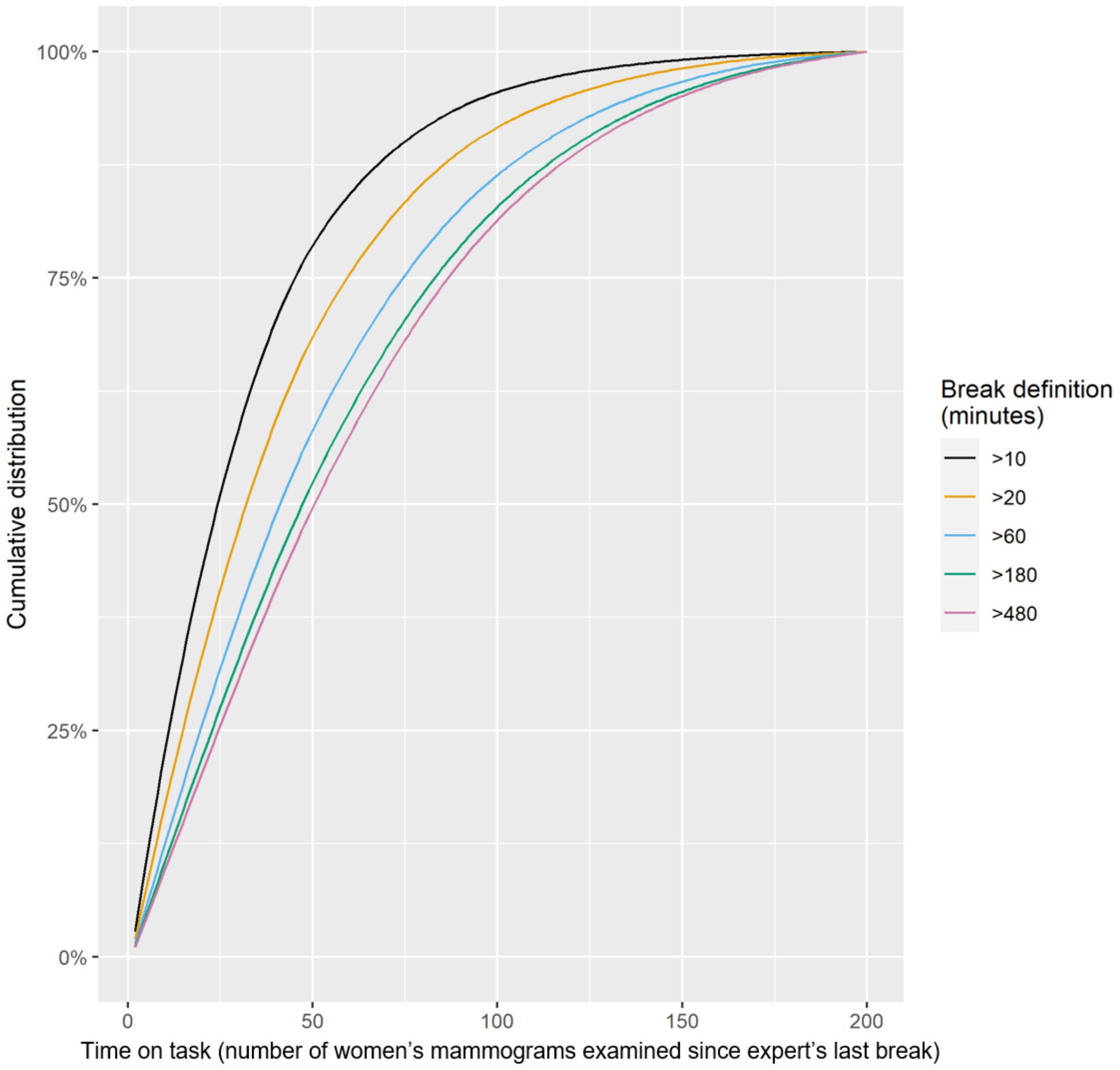

Figure S1. Cumulative distribution of women in the dataset by number of women's mammograms examined since experts last break (from 2 to 200). Includes only those examined in the intended order and with woman's age present in the dataset. Shown for all values of the break definition. The total number of mammograms read for each break definition is shown in row 2 of Table S1.

2. Supplementary tables - Descriptive statistics

The exclusion of mammograms from the dataset due to missing data to identify the expert (71,695) or due to the first expert not examining in the intended order (a further 52,886) reduced the dataset to 1,069,566 women's mammograms, of which 37 were a second set of mammograms from an individual woman.

The number of women included at each session position is dependent on the session definition and is given in figure S1. Using the first expert only, the recall rate was 4.8% and the cancer detection rate 7.4 per thousand women screened.

There were 410 experts in the study identified by their pseudonymised login at the computer system at each breast screening centre. Of these, only 360 pseudonymised codes were unique across the whole dataset, with the same pseudonymised code appearing at more than one centre. It was not possible to identify whether the same expert worked at two different breast screening centres or whether the same login was a coincidence, so we conservatively report only 360 readers.

Descriptive statistics of the three outcomes under the different thresholds are shown in Table S1.

Table S1. Descriptive statistics of the outcomes for each threshold.

	Definition of a break (time without inputting a decision into the software)				
	≥10 minutes	≥20 minutes	≥60minutes	≥180minutes	≥480 minutes
Number of women's mammograms (position 2-200)	1,037,145	1,042,597	1,040,970	1,035,758	1,034,031
Number of women's mammograms (position 2-200, age recorded)	1,037,140	1,042,592	1,040,965	1,035,753	1,034,026
Recall rate	4.63%	4.73%	4.77%	4.78%	4.79%
(women recalled/total women)	(47,993/1,307,140)	(49,344/1,042,592)	(49,610/1,040,965)	(49,548/1,035,753)	(49,508/1,034,026)
Cancer detection rate	0.726%	0.740%	0.745%	0.745%	0.746%
(women with cancer detected/total women)	(7,529/1,307,140)	(7,715/1,042,592)	(7,757/1,040,965)	(7,717/1,035,753)	(7,709/1,034,026)
Number of women's mammograms (position 2-200, age recorded, time between 1s and 600s inclusive)	1037135	1033433	1025888	1018158	1015296
Median time taken (inter-quartile range, s)	35 (19 - 69)	35 (19-69)	35 (20 – 69)	35 (20 – 69)	35 (20 – 70)
Interval cancers per 1,000 women	1.90	1.91	1.90	1.91	1.91
(women with interval cancer within 3 years / number of women)	(1,975/1,037,140)	(1,987/1,042,592)	(1,980/1,040,965)	(1,974/1,035,753)	(1,975/1,034,026)

3. Supplementary tables - Describing the models

The model coefficients for the main model adjusted for the woman's age and whether she had previously attended are given in table S2, with fitted values in table S3. The coefficients of an unadjusted but otherwise equivalent model are given in table S4, with fitted values in table S5.

Table S2. Model coefficients (with 95% confidence intervals) and random effect standard deviations for models for the three outcomes at the different break definitions. For recall and cancer detection the coefficients from their logistic models are shown as odds ratios (OR). For time taken to read the coefficients are shown on the linear scale. The models for recall and time taken used a linear basis spline for session position, with knots at positions 20 and 40. Instead of the model coefficients the gradient of those three lines is shown; as an OR for recall, and linearly for time taken. The gradient is over five session positions, rather than one. Age was standardised in the models. The coefficients shown in these tables have been adjusted (divided by the standard deviation, before conversion to the odds ratio scale) to show the effect of an increase of one year on the outcome. The intercept terms should be interpreted as the outcome at session position two for a mammogram of the mean age that is incident (not a woman's first mammogram). The model results for prevalent (woman's first mammogram) rather than incident are shown in the tables. The standard deviation of the random effects at each level are abbreviated to "RE SD". The cancer detection and recall results are shown to three decimal places (except for the cancer detection intercept and session position) and the time taken results to three significant digits.

	**Definition of a break (time without inputting a decision into the software)**				
	**≥10 minutes**	**≥20 minutes**	**≥60minutes**	**≥180minutes**	**≥480 minutes**
**Cancer Detection Rate**					
Intercept (Position 2)	0.00603	0.00631	0.00629	0.00628	0.00627
95% CI	(0.00572 - 0.00635)	(0.00599 - 0.00665)	(0.00597 - 0.00663)	(0.00595 - 0.00663)	(0.00594 - 0.00662)
Session Position (OR over five positions)	1.00070	0.99755	0.99899	0.99922	0.99937
95% CI	(0.99687 - 1.00454)	(0.99434 - 1.00078)	(0.99617 - 1.00182)	(0.99656 - 1.00189)	(0.99675 - 1.00199)
Age (OR)	1.052	1.052	1.052	1.052	1.052
95% CI	(1.048 - 1.056)	(1.048 - 1.056)	(1.048 - 1.056)	(1.048 - 1.056)	(1.048 - 1.056)
Prevalent (OR)	1.763	1.738	1.741	1.736	1.739
95% CI	(1.640 - 1.895)	(1.618 - 1.867)	(1.621 - 1.870)	(1.616 - 1.865)	(1.619 - 1.868)
RE SD - Centre	0.143	0.145	0.139	0.140	0.142
RE SD - Expert	0.100	0.096	0.097	0.098	0.097

**Recall Rate**					
Intercept (Position 2)	0.036	0.038	0.040	0.040	0.040
95% CI	(0.032 - 0.040)	(0.035 - 0.042)	(0.036 - 0.044)	(0.036 - 0.044)	(0.037 - 0.045)
Session Position 2-20 (OR over five positions)	0.974	0.973	0.970	0.973	0.970
95% CI	(0.964 - 0.984)	(0.962 - 0.984)	(0.958 - 0.982)	(0.960 - 0.986)	(0.957 - 0.983)
Session Position 20-40 (OR over five positions)	0.989	0.984	0.981	0.978	0.979
95% CI	(0.981 - 0.997)	(0.977 - 0.992)	(0.973 - 0.989)	(0.970 - 0.986)	(0.971 - 0.988)
Session Position 40-200 (OR over five positions)	0.994	0.993	0.994	0.995	0.994
95% CI	(0.991 - 0.997)	(0.991 - 0.995)	(0.992 - 0.995)	(0.993 - 0.996)	(0.993 - 0.996)
Age (OR)	1.004	1.005	1.005	1.005	1.005
95% CI	(1.003 - 1.006)	(1.003 - 1.006)	(1.003 - 1.006)	(1.003 - 1.006)	(1.003 - 1.006)
Prevalent (OR)	2.739	2.717	2.716	2.713	2.713
95% CI	(2.665 - 2.815)	(2.645 - 2.791)	(2.644 - 2.789)	(2.641 - 2.787)	(2.641 - 2.787)
RE SD - Centre	0.338	0.339	0.335	0.334	0.334
RE SD - Expert	0.303	0.298	0.299	0.298	0.297
**Time taken to read (s)**					
Intercept (Position 2)	72.3	73.3	74.9	76	76.5
95% CI	(72.1 - 72.6)	(73.1 - 73.5)	(74.6 - 75.2)	(75.6 - 76.5)	(76.2 - 76.8)
Session Position 2-20 (Over five positions)	-2.66	-2.58	-2.72	-2.91	-2.97
95% CI	(-2.71 - -2.61)	(-2.63 - -2.53)	(-2.77 - -2.67)	(-2.97 - -2.85)	(-3.02 - -2.91)
Session Position 20-40 (Over five positions)	0.0552	-0.101	-0.226	-0.234	-0.245
95% CI	(0.00471 - 0.106)	(-0.15 - -0.0522)	(-0.277 - -0.175)	(-0.291 - -0.176)	(-0.302 - -0.189)
Session Position 40-200 (Over five positions)	-0.243	-0.276	-0.295	-0.284	-0.284
95% CI	(-0.253 - -0.234)	(-0.283 - -0.269)	(-0.304 - -0.287)	(-0.293 - -0.275)	(-0.291 - -0.276)
Age (OR)	0.0473	0.047	0.0503	0.0498	0.048
95% CI	(0.036 - 0.0586)	(0.0357 - 0.0584)	(0.0387 - 0.0619)	(0.0386 - 0.0611)	(0.0373 - 0.0588)

Prevalent (OR)	1.57	1.59	1.62	1.51	1.46
95% CI	(1.4 - 1.74)	(1.38 - 1.79)	(1.42 - 1.81)	(1.34 - 1.68)	(1.31 - 1.61)
RE SD - Centre	28.9	29	28.8	28.8	28.7
RE SD - Expert	16.3	16.4	16.5	16.6	16.6
RE SD - Observation	1.25	1.25	1.25	1.24	1.24

Table S3. Fitted values for selected session positions from the models of different break definitions, with 95% confidence intervals, for all three outcomes. The session positions listed are chosen throughout the range and include the knot points used in the basis spline of session position used in the models, positions 20 and 40.

	**Definition of a break (time without inputting a decision into the software)**				
	**≥10 minutes**	**≥20 minutes**	**≥60minutes**	**≥180minutes**	**≥480 minutes**
**Cancer Detection Rate (%) at session position**					
2	0.679	0.708	0.707	0.705	0.704
95% CI	(0.646 - 0.712)	(0.675 - 0.744)	(0.673 - 0.743)	(0.670 - 0.742)	(0.669 - 0.741)
20	0.680	0.702	0.704	0.703	0.703
95% CI	(0.651 - 0.711)	(0.672 - 0.734)	(0.673 - 0.737)	(0.672 - 0.736)	(0.671 - 0.736)
40	0.682	0.696	0.701	0.701	0.701
95% CI	(0.654 - 0.712)	(0.667 - 0.725)	(0.673 - 0.732)	(0.672 - 0.732)	(0.672 - 0.732)
60	0.684	0.689	0.699	0.699	0.699
95% CI	(0.652 - 0.717)	(0.660 - 0.719)	(0.670 - 0.729)	(0.670 - 0.729)	(0.671 - 0.729)
100	0.688	0.675	0.693	0.695	0.696
95% CI	(0.644 - 0.735)	(0.638 - 0.715)	(0.659 - 0.729)	(0.662 - 0.729)	(0.664 - 0.729)
150	0.693	0.659	0.686	0.689	0.692
95% CI	(0.628 - 0.765)	(0.608 - 0.715)	(0.640 - 0.736)	(0.646 - 0.736)	(0.649 - 0.737)
200	0.698	0.643	0.679	0.684	0.687
95% CI	(0.610 - 0.798)	(0.576 - 0.718)	(0.618 - 0.746)	(0.627 - 0.746)	(0.631 - 0.748)
**Recall rate (%) at session position**					
2	4.397	4.656	4.839	4.891	4.947
95% CI	(3.990 - 4.843)	(4.230 - 5.123)	(4.391 - 5.330)	(4.436 - 5.390)	(4.486 - 5.454)
20	4.017	4.236	4.363	4.451	4.459
95% CI	(3.646 - 4.424)	(3.851 - 4.659)	(3.963 - 4.801)	(4.043 - 4.898)	(4.050 - 4.907)
40	3.844	3.990	4.057	4.087	4.116

95% CI	(3.489 - 4.234)	(3.628 - 4.386)	(3.689 - 4.460)	(3.717 - 4.492)	(3.744 - 4.523)
60	3.759	3.887	3.958	4.002	4.028
95% CI	(3.414 - 4.138)	(3.537 - 4.271)	(3.601 - 4.349)	(3.641 - 4.396)	(3.666 - 4.424)
100	3.595	3.690	3.767	3.836	3.858
95% CI	(3.257 - 3.966)	(3.354 - 4.059)	(3.426 - 4.142)	(3.490 - 4.215)	(3.511 - 4.239)
150	3.399	3.458	3.541	3.638	3.656
95% CI	(3.052 - 3.784)	(3.125 - 3.824)	(3.208 - 3.907)	(3.300 - 4.009)	(3.318 - 4.027)
200	3.213	3.239	3.328	3.450	3.463
95% CI	(2.843 - 3.631)	(2.900 - 3.617)	(2.996 - 3.696)	(3.113 - 3.822)	(3.128 - 3.834)
**Time taken to read (s) at session position**					
2	72.7	73.7	75.3	76.4	76.8
95% CI	(72.4 - 72.9)	(73.4 - 73.9)	(74.9 - 75.6)	(75.9 - 76.8)	(76.5 - 77.1)
20	63.1	64.4	65.5	65.9	66.1
95% CI	(62.8 - 63.4)	(64.1 - 64.6)	(65.1 - 65.9)	(65.4 - 66.4)	(65.8 - 66.4)
40	63.3	64.0	64.6	64.9	65.1
95% CI	(63.0 - 63.6)	(63.7 - 64.2)	(64.2 - 64.9)	(64.4 - 65.4)	(64.8 - 65.4)
60	62.3	62.9	63.4	63.8	64.0
95% CI	(62.1 - 62.6)	(62.6 - 63.1)	(63.0 - 63.7)	(63.3 - 64.3)	(63.7 - 64.3)
100	60.4	60.6	61.0	61.5	61.7
95% CI	(60.1 - 60.7)	(60.4 - 60.9)	(60.7 - 61.4)	(61.0 - 62.0)	(61.4 - 62.0)
150	58.0	57.9	58.1	58.7	58.9
95% CI	(57.7 - 58.3)	(57.6 - 58.1)	(57.7 - 58.4)	(58.2 - 59.2)	(58.6 - 59.2)
200	55.5	55.1	55.1	55.8	56.0
95% CI	(55.2 - 55.9)	(54.9 - 55.4)	(54.7 - 55.5)	(55.3 - 56.4)	(55.7 - 56.4)

Table S4. Model coefficients (with 95% confidence intervals) and random effect standard deviations for models for the three outcomes at the different break definitions for sessions, which have not been adjusted for age and prevalence status. For recall and cancer detection the coefficients from their logistic models are shown as odds ratios (OR). For time taken to read the coefficients are shown on the linear scale. The models for recall and time taken used a linear basis spline for session position, with knots at positions 20 and 40. Instead of the model coefficients the gradient of those three lines is shown; as an OR for recall, and linearly for time taken. The gradient is over five session positions, rather than one. The intercept terms should be interpreted as the outcome at session position two. The standard deviation of the random effects at each level are abbreviated to "RE SD". The cancer detection and recall results are shown to three decimal places (except for the cancer detection intercept and session position) and the time taken results to three significant digits.

	**Definition of a break (time without inputting a decision into the software)**				
	**≥10 minutes**	**≥20 minutes**	**≥60minutes**	**≥180minutes**	**≥480 minutes**
**Cancer Detection Rate**					
Intercept (Position 2)	0.00712	0.00743	0.00742	0.00740	0.00739
95% CI	(0.00679 - 0.00747)	(0.00709 - 0.00779)	(0.00707 - 0.00779)	(0.00704 - 0.00777)	(0.00703 - 0.00777)
Session Position (OR over five positions)	1.00044	0.99752	0.99893	0.99919	0.99936
95% CI	(0.99662 - 1.00427)	(0.99431 - 1.00074)	(0.99611 - 1.00175)	(0.99653 - 1.00186)	(0.99675 - 1.00198)
RE SD – Centre	0.143	0.145	0.140	0.141	0.143
RE SD – Expert	0.096	0.092	0.093	0.094	0.093
**Recall Rate**					
Intercept (Position 2)	0.048	0.051	0.053	0.053	0.054
95% CI	(0.044 - 0.053)	(0.046 - 0.056)	(0.048 - 0.058)	(0.048 - 0.059)	(0.049 - 0.059)
Session Position 2-20 (OR over five positions)	0.968	0.966	0.963	0.966	0.963
95% CI	(0.958 - 0.978)	(0.956 - 0.977)	(0.951 - 0.975)	(0.953 - 0.979)	(0.951 - 0.977)
Session Position 20-40 (OR over five positions)	0.989	0.985	0.982	0.98	0.981

95% CI	(0.981 - 0.997)	(0.978 - 0.993)	(0.974 - 0.990)	(0.972 - 0.988)	(0.973 - 0.990)
Session Position 40-200 (OR over five positions)	0.996	0.994	0.995	0.995	0.995
95% CI	(0.993 - 0.999)	(0.992 - 0.997)	(0.993 - 0.996)	(0.993 - 0.997)	(0.993 - 0.996)
RE SD - Centre	0.336	0.337	0.334	0.333	0.333
RE SD - Expert	0.308	0.302	0.303	0.301	0.300
**Time taken to read (s)**					
Intercept	72.7	73.7	75.3	76.4	76.8
95% CI	(72.5 - 72.9)	(73.5 - 73.9)	(75 - 75.6)	(76.1 - 76.6)	(76.4 - 77.2)
Session Position 2-20 (Over five positions)	-2.66	-2.59	-2.72	-2.92	-2.97
95% CI	(-2.7 - -2.62)	(-2.63 - -2.54)	(-2.78 - -2.67)	(-2.97 - -2.86)	(-3.02 - -2.92)
Session Position 20-40 (Over five positions)	0.053	-0.101	-0.228	-0.234	-0.246
95% CI	(0.0102 - 0.0957)	(-0.146 - -0.056)	(-0.283 - -0.172)	(-0.289 - -0.18)	(-0.304 - -0.188)
Session Position 40-200 (Over five positions)	-0.243	-0.276	-0.295	-0.284	-0.284
95% CI	(-0.25 - -0.237)	(-0.284 - -0.269)	(-0.303 - -0.287)	(-0.292 - -0.276)	(-0.292 - -0.275)
RE SD - Centre	28.9	29	28.8	28.8	28.7
RE SD - Expert	16.3	16.4	16.5	16.6	16.6
RE SD - Observation	1.25	1.25	1.25	1.24	1.24

Table S5. Fitted values for selected session positions from the models of different thresholds not adjusted for age and prevalence status, with 95% confidence intervals, for all three outcomes. The session positions listed are chosen throughout the range and include the knot points used in the basis spline of session position used in the recall and time taken models, positions 20 and 40.

	**Definition of a break (time without inputting a decision into the software)**				
	**≥10 minutes**	**≥20 minutes**	**≥60minutes**	**≥180minutes**	**≥480 minutes**
**Cancer Detection Rate (%) at session position**					
2	0.712	0.743	0.742	0.740	0.739
95% CI	(0.679 - 0.747)	(0.709 - 0.779)	(0.707 - 0.779)	(0.704 - 0.777)	(0.703 - 0.777)
20	0.713	0.737	0.739	0.738	0.737
95% CI	(0.684 - 0.744)	(0.706 - 0.768)	(0.708 - 0.772)	(0.705 - 0.771)	(0.705 - 0.771)
40	0.715	0.729	0.736	0.735	0.735
95% CI	(0.686 - 0.745)	(0.701 - 0.759)	(0.707 - 0.766)	(0.705 - 0.766)	(0.706 - 0.767)
60	0.716	0.722	0.733	0.733	0.734
95% CI	(0.684 - 0.750)	(0.693 - 0.753)	(0.704 - 0.763)	(0.704 - 0.763)	(0.705 - 0.764)
100	0.718	0.708	0.727	0.728	0.730
95% CI	(0.673 - 0.767)	(0.670 - 0.748)	(0.692 - 0.763)	(0.695 - 0.763)	(0.697 - 0.764)
150	0.722	0.691	0.719	0.722	0.725
95% CI	(0.654 - 0.796)	(0.637 - 0.749)	(0.671 - 0.770)	(0.677 - 0.770)	(0.681 - 0.772)
200	0.725	0.674	0.711	0.717	0.721
95% CI	(0.634 - 0.828)	(0.604 - 0.752)	(0.648 - 0.781)	(0.657 - 0.781)	(0.663 - 0.784)
**Recall rate (%) at session position**					
2	4.806	5.082	5.287	5.338	5.394
95% CI	(4.362 - 5.293)	(4.618 - 5.590)	(4.798 - 5.822)	(4.844 - 5.879)	(4.896 - 5.940)
20	4.296	4.518	4.648	4.740	4.749
95% CI	(3.899 - 4.732)	(4.106 - 4.969)	(4.222 - 5.115)	(4.306 - 5.215)	(4.315 - 5.224)
40	4.115	4.270	4.343	4.387	4.420

95% CI	(3.734 - 4.533)	(3.882 - 4.694)	(3.948 - 4.775)	(3.991 - 4.820)	(4.023 - 4.855)
60	4.049	4.180	4.253	4.303	4.333
95% CI	(3.676 - 4.457)	(3.803 - 4.593)	(3.868 - 4.673)	(3.917 - 4.727)	(3.945 - 4.757)
100	3.919	4.007	4.078	4.141	4.163
95% CI	(3.552 - 4.324)	(3.641 - 4.408)	(3.707 - 4.483)	(3.768 - 4.550)	(3.789 - 4.571)
150	3.764	3.800	3.869	3.947	3.959
95% CI	(3.381 - 4.188)	(3.435 - 4.202)	(3.505 - 4.269)	(3.581 - 4.348)	(3.595 - 4.359)
200	3.614	3.603	3.670	3.761	3.766
95% CI	(3.200 - 4.078)	(3.227 - 4.021)	(3.304 - 4.075)	(3.394 - 4.165)	(3.402 - 4.166)
**Time taken to read (s) at session position**					
2	72.7	73.7	75.3	76.4	76.8
95% CI	(72.5 - 72.9)	(73.5 - 73.9)	(75.0 - 75.6)	(76.1 - 76.6)	(76.4 - 77.2)
20	63.1	64.4	65.5	65.9	66.1
95% CI	(62.9 - 63.3)	(64.1 - 64.6)	(65.1 - 65.8)	(65.5 - 66.2)	(65.7 - 66.6)
40	63.3	64.0	64.6	64.9	65.1
95% CI	(63.1 - 63.5)	(63.7 - 64.2)	(64.2 - 64.9)	(64.6 - 65.2)	(64.7 - 65.5)
60	62.3	62.9	63.4	63.8	64.0
95% CI	(62.1 - 62.6)	(62.6 - 63.1)	(63.1 - 63.7)	(63.5 - 64.1)	(63.6 - 64.4)
100	60.4	60.6	61.0	61.5	61.7
95% CI	(60.2 - 60.6)	(60.4 - 60.9)	(60.7 - 61.4)	(61.3 - 61.8)	(61.3 - 62.2)
150	58.0	57.9	58.1	58.7	58.9
95% CI	(57.8 - 58.2)	(57.6 - 58.1)	(57.7 - 58.4)	(58.4 - 59.0)	(58.4 - 59.4)
200	55.5	55.1	55.1	55.8	56.0
95% CI	(55.3 - 55.8)	(54.8 - 55.4)	(54.7 - 55.5)	(55.6 - 56.1)	(55.5 - 56.6)

66

4. Median speed of reading

The primary measure of time taken to examine each case was a mean, with cases taking longer than 10 minutes excluded so that the mean was not overly influenced by the tail of the distribution. The median time taken with no exclusions is shown in figure S2. This demonstrates the same pattern of decreasing time taken per case with increasing time on task.

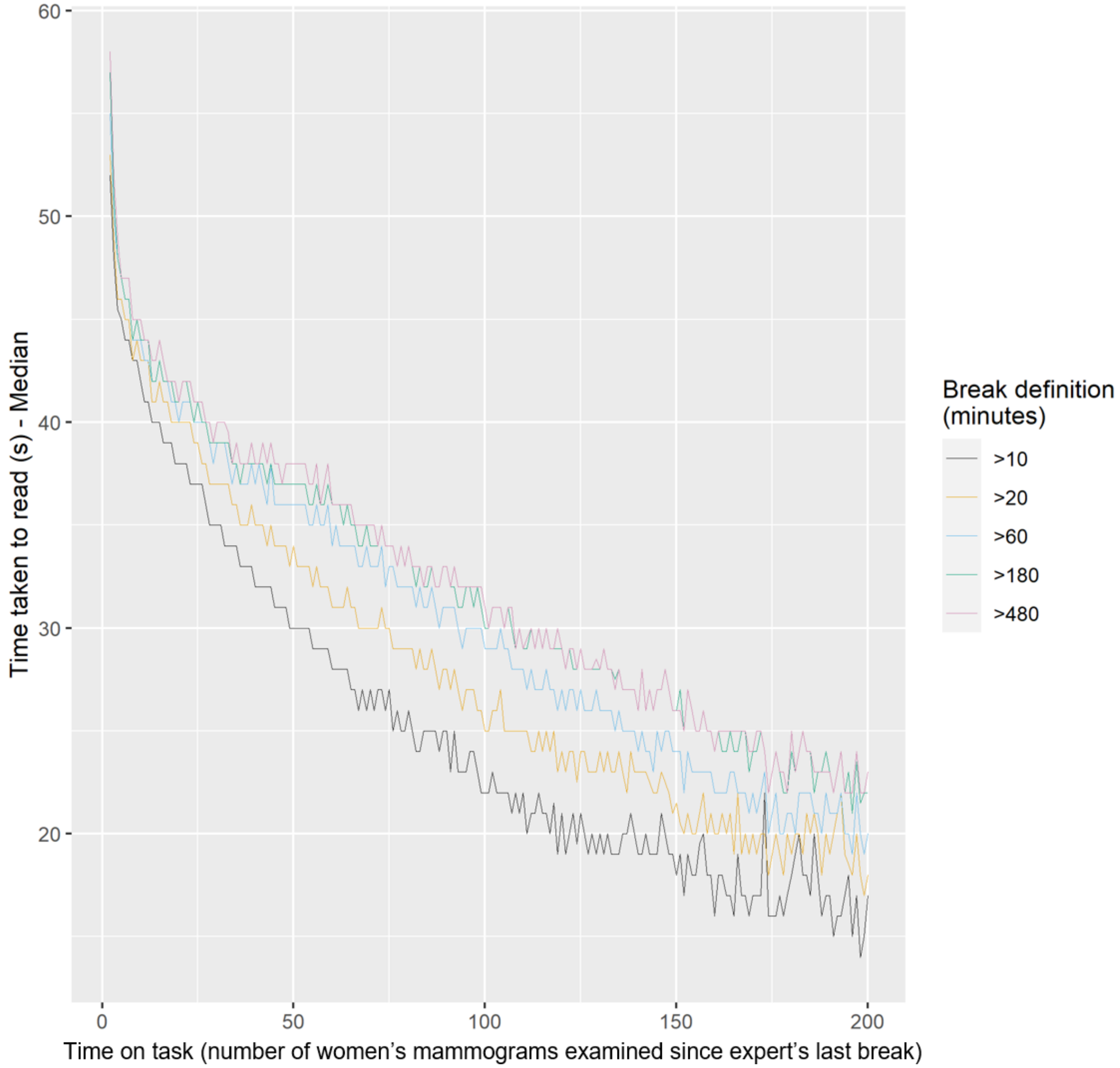

Figure S2. Median time taken to read for each position in a reading session, by break definition. Mammograms read in order only, excluding those with time taken to read of zero, but with no upper limit on time taken.

5. Models including cases examined out of intended order

The main models exclude cases which were not examined in the intended order, so that experts coming back to more difficult cases later could not bias results. Models were repeated including these cases examined out of the intended order and it did not affect results, as shown in figure S3.

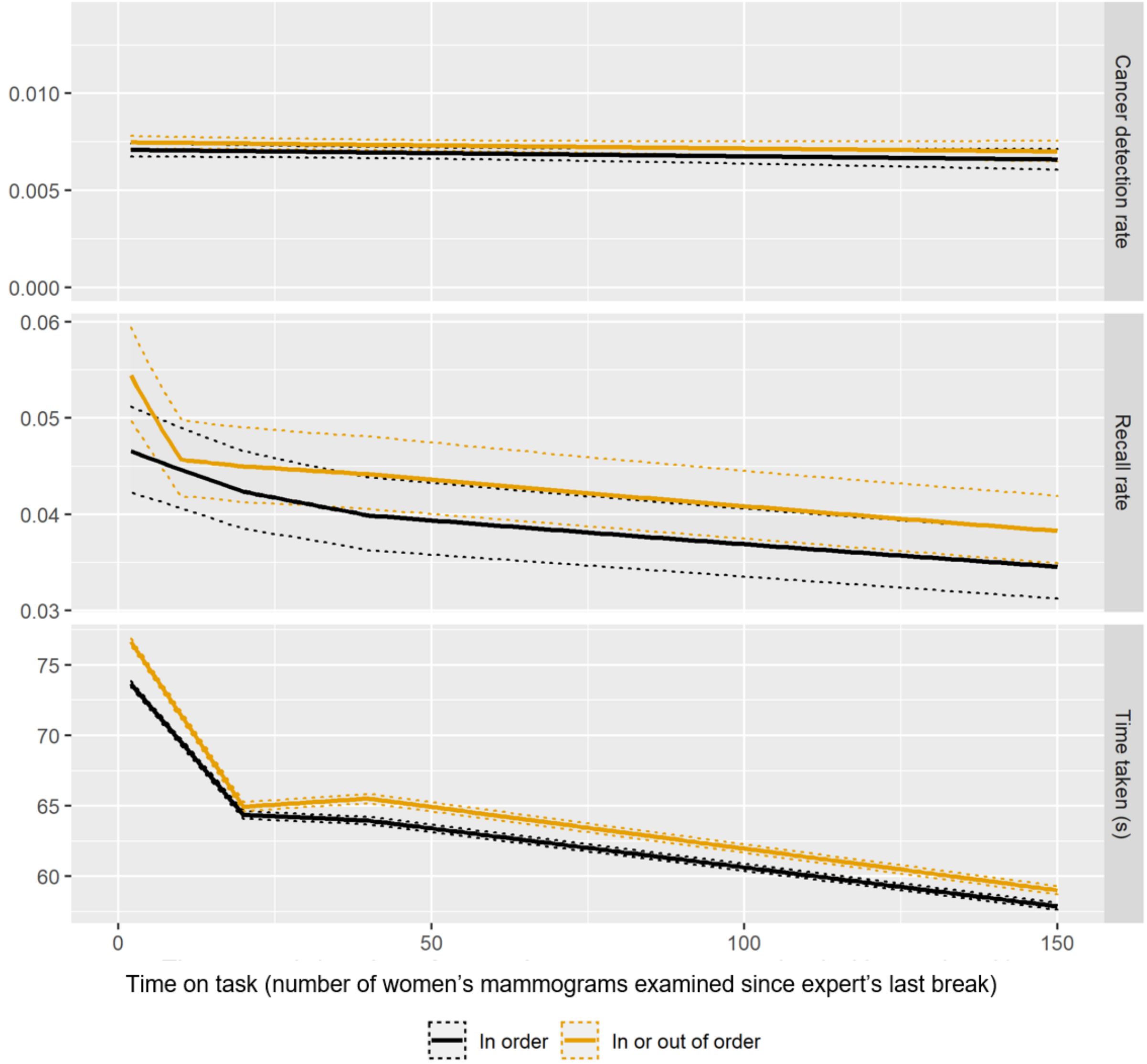

Figure S3. Model fitted values (shown as a solid line with 95% confidence intervals as dotted lines) including (orange) and excluding (black) women's mammograms examined in a different order to that intended. Performance metrics with time on task: cancer detection rate calculated as the proportion of women in which cancer was detected by the expert; recall rate calculated as the proportion of women that the expert indicated required recall for further tests; and mean time

taken to examine each woman's mammograms. Time on task is represented by the number of women's mammograms examined consecutively without a break. Break defined as 20 minutes without inputting a decision on the computer. Models were adjusted for women's age and whether she has previously attended screening, with clustering for expert and screening centre.

6. Break duration

To investigate the possible interaction between the length of the reader's breaks and the vigilance decrement/ improvement, outcomes for sessions starting after a short break, a moderate break, or a long break were plotted. This demonstrates that specificity and criterion are lower after a long break of >12hours, compared to after a short break of <1hour.

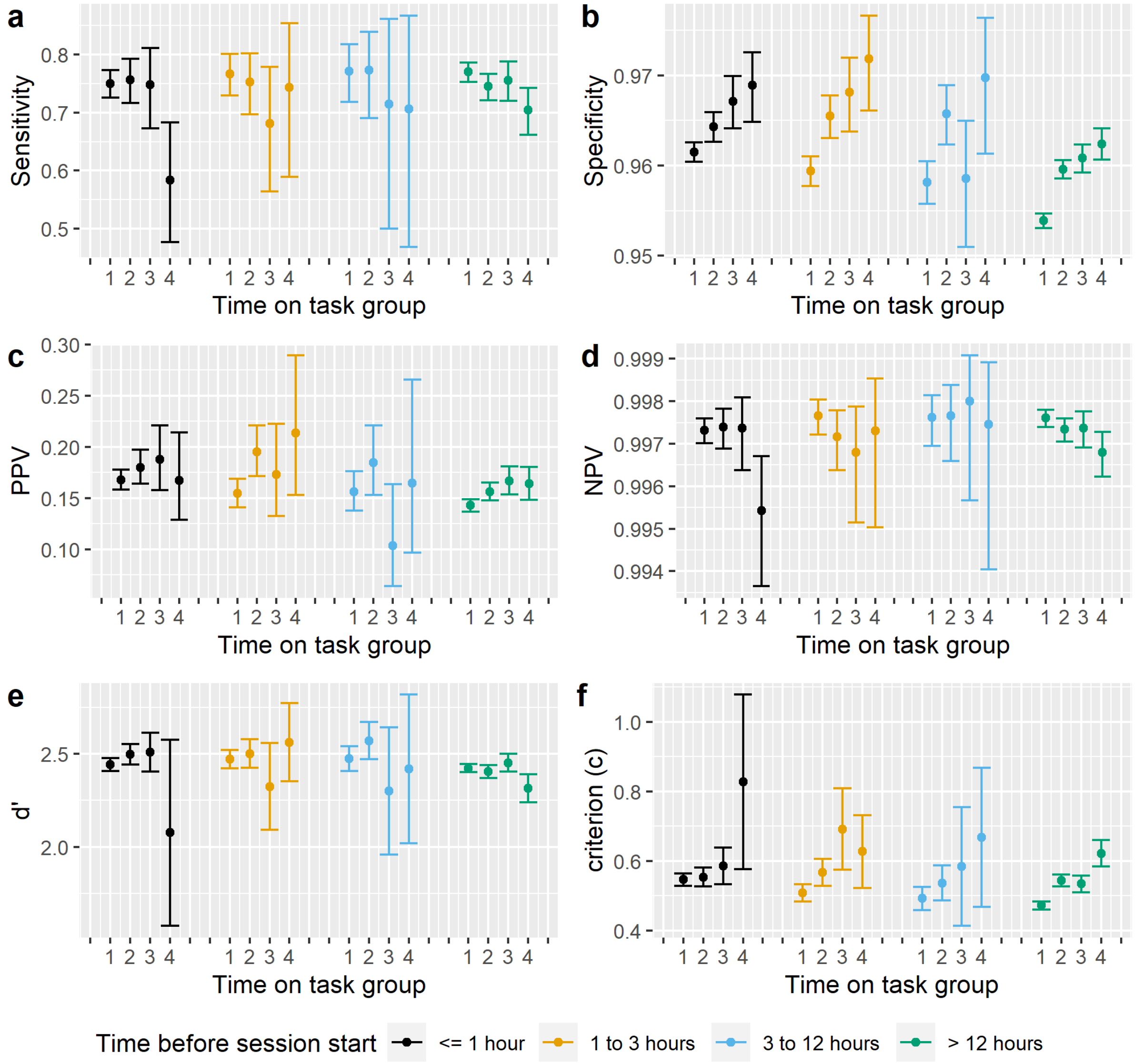

Figure S4. Outcomes for sessions starting after a short break of <1hour, a moderate break of 1 to 3 or 3 to 12 hours, or a long break of >12 hours which represents the next working day. The session is considred ended after a break without entering an opinion of more than 20 minutes in all definitions.

7. Time taken to read

To investigate the difference between the mean and median time taken to examine each woman's mammogram, the time taken to read was plotted using a 20-minute threshold:

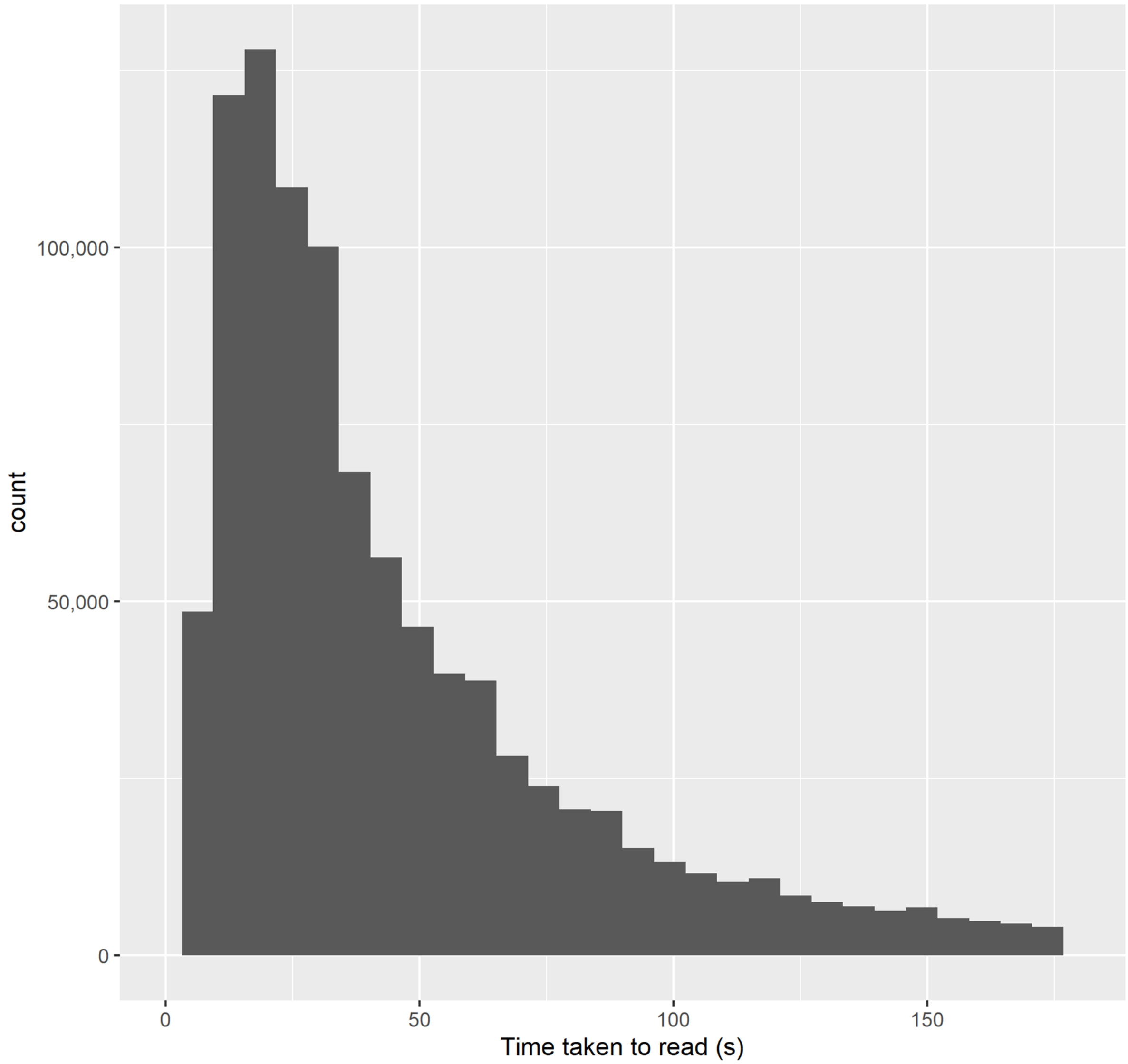

Figure S5. Histogram of time taken to read, using 20 minute threshold, horizontal axis limited to 180s.
